# Effect of parallel adolescent and parent dialectical behavior therapy skills training vs. adolescent-only skills training on nonsuicidal self-injury: a pilot randomized trial

**DOI:** 10.1186/s12888-026-08199-3

**Published:** 2026-06-01

**Authors:** Shunhua Ye, Xueling Wei, Chen Deng, Peiyi Chen, Chanjuan Yang, Yanling Zhou, Jianghui Dong

**Affiliations:** 1 The Affiliated Brain Hospital, Guangzhou Medical University, Guangzhou, China; 2 Guangdong Engineering Technology Research Center for Translational Medicine of Mental Disorders, Guangzhou, China; 3 The Born in Guangzhou Cohort Study Group, Department of Pediatrics, Guangzhou Women and Children’s Medical Center, Guangzhou Medical University, Guangzhou, China

**Keywords:** Nonsuicidal self-injury, Dialectical behavior therapy, Adolescents skills training, Parent involvement, Randomized controlled trial, Family interaction

## Abstract

**Background:**

Family involvement in dialectical behavior therapy (DBT) improves nonsuicidal self-injury (NSSI) outcomes in adolescents, yet many adolescents resist conjoint treatment with parents, highlighting the need for alternative delivery formats. This pilot study preliminarily examined whether parallel adolescent and parent DBT skills training (DBT-SP, in which parents attended separate concurrent skills groups) shows promise compared with adolescent-only DBT skills training (DBT-S) in reducing NSSI and associated symptoms.

**Methods:**

In this pilot randomized controlled trial, 38 adolescents aged 12–18 years meeting DSM-5 criteria for NSSI disorder were randomized to DBT-SP (*n* = 19) or DBT-S (*n* = 19). Adolescents in both groups received weekly 120-minute group sessions over 12 weeks. Additionally, parents in the DBT-SP group received parallel 120-minute skills training sessions (separate from adolescents), while parents in the DBT-S group received no structured intervention. The primary outcome was NSSI severity assessed using the Ottawa Self-Injury Inventory at baseline and at weeks 4, 8, and 12.

**Results:**

NSSI frequency decreased in both groups from baseline to week 12 (time effect, *p* < 0.01). The DBT-SP group demonstrated greater reductions in depressive (*p* = 0.03) and anxiety symptoms (*p* = 0.01). Positive parent-adolescent interactions increased from a median of 2 at baseline to 11 times weekly by the end of week 11 in the DBT-SP group while remaining stable in the DBT-S group (interaction *p* < 0.01).

**Conclusions:**

In this pilot randomized controlled trial, compared with adolescent-only delivery, parallel adolescent-parent DBT skills training was associated with reduced anxiety and depressive symptoms in adolescents and increased positive family interactions. These preliminary findings warrant replication in larger, fully powered confirmatory trials.

**Clinical trial number:**

Chinese Clinical Trial Registry, ChiCTR2500105036. Retrospectively registered on June 27, 2025.

**Supplementary Information:**

The online version contains supplementary material available at 10.1186/s12888-026-08199-3.

## Introduction

Non-suicidal self-injury (NSSI) has emerged as a major global public health concern [1]. A global meta-analysis estimated a lifetime prevalence of 17.2% and a 12-month prevalence of 13.4% for NSSI in community samples of adolescents [2]. The situation in China appears similarly concerning: a meta-analysis encompassing nearly 150,000 secondary school students reported a pooled NSSI prevalence of 27.4%, with evidence of a year-on-year increase [3]. Beyond direct physical harm, NSSI has been closely associated with substantial psychosocial impairment, high-frequency utilization of healthcare services, and considerable family burden [1, 4, 5].

The clinical significance of NSSI also lies in its status as a transdiagnostic phenotype that is closely intertwined with affective disorders and represents one of the strongest predictors of future suicidal behavior [6, 7]. Over 70% of adolescents who engage in NSSI meet criteria for at least one psychiatric disorder, with major depressive disorder co-occurring in 50–70% of cases [8]. Longitudinal studies have consistently shown that adolescents with a history of NSSI are at 5 - to 10 - fold greater risk of suicide attempts compared with those without such a history [8, 9]. Effective intervention for NSSI therefore requires early engagement with the underlying mechanisms of affective dysregulation [10].

Affective dysregulation has been identified as a major maintaining mechanism of NSSI, and dialectical behavior therapy (DBT) - which systematically targets emotion regulation capacity - is among the most empirically supported interventions for this population [11, 12]. Through its four core modules of mindfulness, distress tolerance, emotion regulation, and interpersonal effectiveness, DBT addresses several functional underpinnings of NSSI [13]. However, comprehensive DBT is resource-intensive and limited in accessibility, which has driven the development of the group-based DBT skills training (DBT-ST) format [9, 11, 14]. Meta-analytic evidence suggests that DBT-ST produces small-to-moderate effect sizes in reducing NSSI frequency among adolescents [3, 15, 16].

In recent years, the research focus has expanded from the individual to the family ecological system in which the adolescent is embedded. A growing body of evidence has identified family conflict, ineffective communication, and parental emotional difficulties as recurrent risk factors for adolescent NSSI [17–20]. Accordingly, incorporating the family into the DBT framework has been proposed as an important augmentation strategy. Family-involved DBT for adolescents, in which parents and adolescents jointly participate in multifamily skills groups - has been associated in multiple studies with reductions in self-injurious behavior and improvements in family functioning [11, 17–19]. Nevertheless, existing family involvement models have notable limitations. First, in multifamily group settings, open discussion of difficulties by high-conflict families may elicit shame and opposition, paradoxically increasing dropout risk [21, 22]. Second, adolescent reluctance to participate in conjoint treatment with parents, coupled with the logistical challenges of coordinating family schedules, further constrains routine implementation [23]. Bridging the gap between efficacy and feasibility may require alternative intervention formats.

One promising approach is the “parallel-concurrent” DBT group treatment format, in which parents and adolescents attend simultaneous but separate DBT skills groups in different physical spaces within the same treatment cycle [24]. This format aims to balance the systemic benefits of family intervention with adolescents’ developmental need for autonomy, fostering a consistent environment for skill application within the family while respecting psychological boundaries, and may thereby reduce barriers to participation [24, 25]. Mechanistic considerations further suggest that when parents concurrently acquire emotion regulation skills, they may more effectively serve as skills coaches in everyday family life, supporting the generalization and maintenance of adolescents’ skills [26, 27].

Building on this line of work, we sought to examine whether the parallel-concurrent format might offer a workable approach for delivering DBT skills training to adolescents and their caregivers in routine practice. Inspired by the theoretical considerations outlined above, we conducted the DBT for Adolescents and Parents in Parallel (DBT-SP) study, a 12-week pilot randomized controlled trial comparing DBT-SP with adolescent-only DBT skills training (DBT-S) on NSSI behaviors, emotion regulation, and family functioning. The primary outcome was defined as the severity of nonsuicidal self-injury (NSSI) behaviors. Secondary outcomes included depressive symptoms, anxiety symptoms, suicidal ideation, emotion regulation abilities, and parent-adolescent interaction and family functioning. Our primary hypothesis was that DBT-SP would lead to greater improvements in NSSI severity compared with DBT-S. Secondary hypotheses and exploratory analyses were conducted for all other outcomes in a hierarchical manner to generate evidence for future confirmatory trials. This trial was designed as a pilot exploratory study aimed primarily at estimating treatment effect sizes, examining the feasibility of the parallel-concurrent format in our setting, and informing the design of future adequately powered confirmatory trials - rather than at supporting definitive inferential claims about the comparative efficacy of the two formats.

## Methods

### Design

#### Study design and ethics approval

**Fig. 1:**
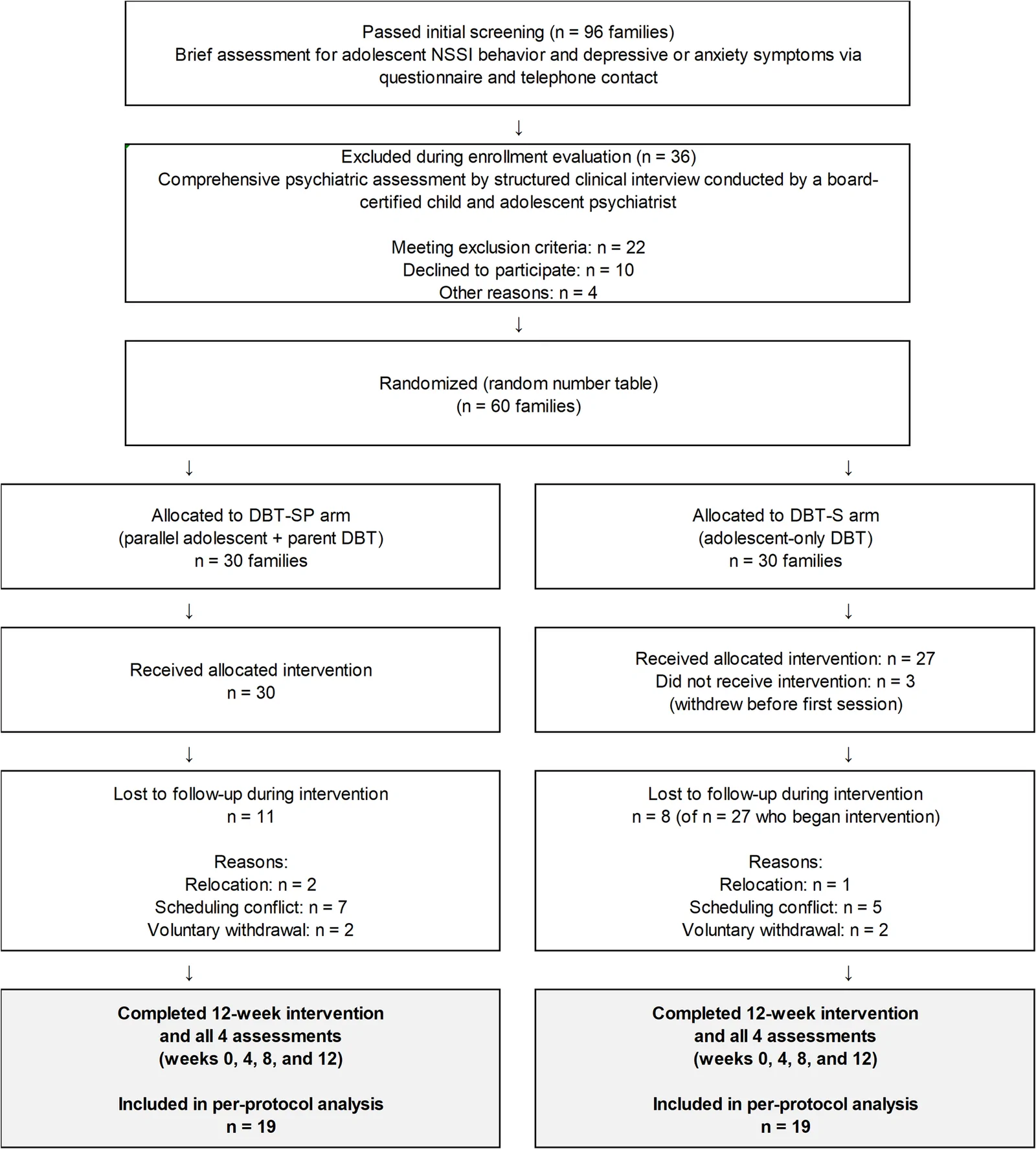
Participant flow diagram (CONSORT)

This study was a single-center, parallel-group pilot randomized controlled trial conducted in a community setting in Guangzhou, China, in 2025. The study protocol, including the pre-specified primary and secondary outcomes and the planned analytic approach, was finalized prior to data collection (protocol version dated April 17, 2025); the trial was subsequently registered with the Chinese Clinical Trial Registry (ChiCTR2500105036) on June 27, 2025. We acknowledge that this registration was retrospective and discuss the implications in the Limitations section. The study was approved by the Ethics Committee of the Affiliated Brain Hospital, Guangzhou Medical University (approval [AF/SC-08/02.3]). Written informed consent was obtained from all legal guardians, and assent was obtained from all adolescent participants prior to enrollment. The trial followed the Consolidated Standards of Reporting Trials (CONSORT) guidelines for parallel-group randomized trials, and the participant flow is presented in Fig.1. Its design and hypotheses are consistent with established adolescent DBT adaptations that emphasize caregiver involvement while holding core DBT content constant [25, 28]. The trial was reported in accordance with the Consolidated Standards of Reporting Trials (CONSORT) 2025 guidelines for parallel-group randomized trials. The participant flow diagram is presented in Fig.1.

#### Participants

Adolescents and their caregivers were recruited through the hospital’s official WeChat account and a collaborative platform operated with the local education bureau. Two psychiatrists screened and diagnosed all potential participants (Fig. 1).

Inclusion criteria for adolescents were: (a) meeting Diagnostic and Statistical Manual of Mental Disorders (Fifth Edition) criteria for non-suicidal self-injury (NSSI) disorder; (b) aged 12 to 18 years; (c) current student enrollment in Guangzhou; and (d) both the adolescent and at least one caregiver willing to participate. Exclusion criteria were: (a) severe acute suicide risk or recent severe depressive or anxiety symptoms requiring immediate intervention; (b) inability to communicate effectively; and (c) comorbid personality disorder, developmental disorder, or severe mental illness (e.g., schizophrenia, bipolar disorder). No other comorbid psychiatric disorders were present in the current study sample beyond those specified in the exclusion criteria.

The analysis sample comprised 38 adolescents (*n* = 19 per arm) who completed the 12-week protocol and all four assessment time points. Consistent with the pilot design of this trial, no formal power calculation was performed. This sample size falls within commonly recommended ranges for pilot trials of behavioral interventions (e.g., 12–30 per arm) and was considered adequate for the primary aim of estimating treatment effect sizes to inform the design of future adequately powered confirmatory trials [29].

#### Randomization and interventions

Eligible adolescents were allocated in a 1:1 ratio to either the DBT-SP or DBT-S using a random number table generated prior to enrollment. Randomization was stratified by sex and age and executed by an independent research team member who was not involved in the screening or outcome assessments.

Both arms received an identical 12-week DBT skills program delivered in weekly 120-minute group sessions. The program covered the four standard modules: mindfulness, distress tolerance, emotion regulation, and interpersonal effectiveness (Table 1); the protocol constituted a skills-training-only intervention without individual psychotherapy or between-session telephone coaching. In the DBT-SP arm, adolescents and at least one caregiver attended concurrent parallel skills groups across all 12 sessions in two separate spaces, with caregiver groups emphasizing validation techniques, behavioral coaching, and contingency management strategies to facilitate skill generalization at home. In DBT-S arm, youth participated in a standalone group without concurrent caregiver involvement. All sessions followed a standardized structure comprising didactic instruction, experiential practice (role-plays and behavioral rehearsal), and between-session exercises, with mindfulness training introduced initially and subsequently integrated as a brief opening exercise. Adolescents maintained weekly practice logs to document home-based skill application, and caregivers in the DBT-SP received corresponding worksheets to guide in-home coaching. Clinical risk was systematically monitored at each assessment point with established hospital referral pathways activated for emergent safety concerns, and all program components adhered to the five essential functions of standard DBT implementation guidelines [30].

**Table 1:** DBT skills training group intervention protocol for adolescents and caregivers

Session	Content (Adolescents)	Content (Caregivers)
Week 1	Group establishment, building therapeutic alliance, identifying and labeling emotions	As adolescent group, plus rationale for caregiver involvement, psychoeducation on adolescent NSSI and emotion dysregulation, and introduction to validation as the core caregiver skill
Week 2	Emotion regulation: checking the facts	As adolescent group, plus recognizing and labeling adolescent emotions and distinguishing emotion from behavior in parent-child exchanges
Week 3	Emotion regulation: opposite action; mindfulness practice	As adolescent group, plus managing caregiver reactivity during conflict episodes
Week 4	Mindfulness practice; ABC PLEASE skills (Accumulate Positive Emotions, Build Mastery, Cope Ahead with Difficult Situations; treat PhysicaL illness, balance Eating, avoid mood-Altering substances, balance Sleep, get Exercise)	As adolescent group, plus modeling self-care behaviors and coaching adolescents in ABC PLEASE skills at home
Week 5	Interpersonal effectiveness: DEAR MAN skills (Describe, Express, Assert, Reinforce, stay Mindful, Appear confident, Negotiate); DEAR MAN advanced applications	As adolescent group, plus applying DEAR MAN to parenting requests and case discussion of common parent-adolescent conflict scenarios
Week 6	Interpersonal effectiveness: FAST skills (be Fair, no excessive Apologies, Stick to values, be Truthful); GIVE skills (be Gentle, act Interested, Validate, use an Easy manner)	As adolescent group, plus balancing limits with warmth and non-judgmental communication during disagreement
Week 7	Distress tolerance: crisis survival strategies	As adolescent group, plus recognizing adolescent distress signals and staying regulated during a child’s crisis
Week 8	Distress tolerance: radical acceptance; turning the mind	As adolescent group, plus accepting the adolescent’s emotional experience without endorsing self-harm
Week 9	Distress tolerance: walking the middle path; IMPROVE the moment (Imagery, Meaning, Prayer/quiet time, Relaxation, One thing in the moment, brief Vacation, Encouragement and self-compassion)	As adolescent group, plus dialectical thinking in parenting (acceptance and change) and applying IMPROVE the moment as a caregiver self-care strategy
Week 10	Advanced emotion regulation; family communication skills	As adolescent group, plus setting up home practice routines that reinforce adolescent skill use
Week 11	Comprehensive DBT skills integration and crisis management	As adolescent group, plus safety planning and crisis response at home, and coordinating with the treatment team
Week 12	Review and consolidation: overview of four core modules (mindfulness, emotion regulation, interpersonal effectiveness, distress tolerance); personal skills toolbox and portable emergency coping card; relapse prevention planning and follow-up arrangements	As adolescent group, plus creating a family skills toolbox and emergency response plan from the caregiver perspective

#### Assessment time points and outcomes

Outcome assessments were administered at baseline (week 0), mid-treatment (weeks 4 and 8), and post-treatment (week 12). The primary endpoint was the week-12 post-treatment assessment. Three consecutive unexcused absences during the intervention period were predefined as dropout. The primary outcome was non-suicidal self-injury severity assessed by the adolescent-reported Ottawa Self-Injury Inventory (OSI) [31], which yields indices of NSSI frequency (thoughts and behaviors) and four functional motivation subscales: emotion regulation, interpersonal influence, self-punishment, and anti-dissociation. Higher scores indicate greater NSSI involvement. Secondary outcomes included adolescent-reported measures of depressive symptoms (Patient Health Questionnaire-9 [PHQ-9], range 0–27) [32]; anxiety symptoms (Generalized Anxiety Disorder-7 [GAD-7], range 0–21) [33]; suicidal ideation (Beck Scale for Suicide Ideation [BSS]) [34]; and emotion regulation (Emotion Regulation Scale[ERS] [35], comprising cognitive reappraisal and expressive suppression subscales).

Parent-adolescent interactions were measured weekly using a study-developed checklist in which adolescents reported the frequency of predefined positive and negative interaction behaviors over the prior 7 days (checklist items and scoring instructions are provided in the Supplement). Acceptable internal consistency for both subscales was observed in the present sample. Psychometric properties of the checklist were examined in an independent sample of 210 adolescents prior to this trial. Confirmatory factor analysis supported a two-factor structure consisting of separate positive and negative interaction subscales. Fit indices for the positive interaction subscale were acceptable (*χ²/df* = 2.73; GFI = 0.96; AGFI = 0.92; NFI = 0.92; CFI = 0.95; RMSEA = 0.09), and fit indices for the negative interaction subscale were within acceptable to marginal ranges (*χ²/df* = 4.24; GFI = 0.94; AGFI = 0.87; NFI = 0.90; CFI = 0.92; RMSEA = 0.12). Internal consistency was acceptable for both the positive interaction subscale (Cronbach’s *α* = 0.79) and the negative interaction subscale (Cronbach’s *α* = 0.82). Sampling adequacy was supported by Kaiser-Meyer-Olkin values of 0.81 for both subscales, and Bartlett’s test of sphericity was significant for both subscales (positive: *χ²*(15) = 313.64, *p* < 0.01; negative: *χ²*(15) = 391.32, *p* < 0.01).

Caregiver-reported measures were collected from parents in both arms to permit between-group comparisons of family-level effects, including parental depressive symptoms (PHQ-9), anxiety symptoms (GAD-7), emotion regulation (ERS), family climate (Chinese Family Environment Scale [FES-CV]) [36], and parenting stress (Parenting Stress Index-Short Form [PSI-SF]), which yields a total score and three subscales: parental distress (PD), parent-child dysfunctional interaction (P-CDI), and difficult child (DC). For all scales, higher scores indicate greater symptom severity or stronger endorsement of the measured construct, except for the ERS cognitive reappraisal subscale where higher scores reflect more adaptive regulation.

### Statistical analysis

Continuous variables were presented as means and standard deviations, while categorical variables were presented as frequencies and percentages. Age comparisons between the two groups were conducted using the rank-sum test. Differences in the distribution of sex, educational level, monthly household income, parental marital status, parental educational attainment, clinically diagnosed mental or psychiatric disorders, use of psychotropic medications and history of psychiatric hospitalisation were examined using the chi-squared test. Data were analysed according to the per-protocol principle. Participants who completed the baseline survey were analysed according to their randomly assigned group.

Linear mixed models were used to compare the two groups’ differences in the primary and secondary outcomes. The model included the variables group (DBT-SP vs. DBT-S), time (baseline, week 4, week 8 and trial endpoint) and time×group interaction. The time×group interaction term reflected the differential change between groups from baseline to the trial endpoint. For the primary NSSI outcomes (overdispersed counts), we fitted negative binomial GLMMs with log link, including group, time, and group×time as fixed effects and a random intercept (compound symmetry) for each participant. Missing data were handled under MAR using RSPL without imputation. Descriptive statistics are medians (IQR); group comparisons are based on model‑estimated least‑squares means (back‑transformed). The time×group interaction tests differential change over time. All analyses used SAS PROC GLIMMIX 9.4.

All analyses were performed using SAS version 9.4, with all P values calculated as two-sided. Given the pilot exploratory orientation of the trial, statistical significance is not used as a binary decision rule; instead, results are interpreted in conjunction with effect sizes and the precision of the corresponding confidence intervals.

## Results

In total, 38 adolescent families participated in the present study, including 19 in the DBT-S group and 19 in the DBT-SP group. The mean ages were 14.3 years (SD, 1.6 years) in the DBT-S group and 14.3 years (SD, 1.5 years) in the DBT-SP group, with females comprising the majority in both groups. As shown in Table 2, baseline characteristics—including age, sex, grade level, household income, parental education, and history of mental disorders, medication use, and hospitalizations.

**Table 2:** Demographic characteristics of the children and families at baseline by intervention status

Characteristic	DBT - S group *n* = 19	DBT - SP group *n* = 19	*p*- value
Age, mean ± SD	14.3 ± 1.6	14.3 ± 1.5	0.618
Sex, n (%)			
Girl	17 (89.5)	16 (84.2)	0.631
Boy	2 (10.5)	3 (15.8)	
Educational attainment			
Junior high school	15 (78.9)	14 (73.7)	0.703
High school	4 (21.1)	5 (26.3)	
Monthly per capita household income			
< 5000	5 (26.3)	5 (26.3)	0.654
5000–15,000	10 (52.6)	12 (63.2)	
>15,000	4 (21.1)	2 (10.5)	
Parental marital status			
Married	13 (68.4)	18 (94.7)	0.036
Others	6 (31.6)	1 (5.3)	
Paternal educational attainment			
Below junior high school	3 (15.8)	7 (36.8)	0.166
Highschool	9 (47.4)	4 (21.1)	
College/university or higher	7 (36.8)	8 (42.1)	
Maternal educational attainment			
Below junior high school	0 (0.0)	3 (15.8)	0.192
Highschool	10 (52.6)	9 (47.4)	
College/university or higher	9 (47.4)	7 (36.8)	
Clinically diagnosed mental/psychiatric disorder	16 (84.2)	14 (73.7)	0.426
Use of psychotropic medications	13 (68.4)	10 (52.6)	0. 319
History of psychiatric hospitalization	3 (15.8)	2 (10.5)	0.631

Among adolescents, both the DBT-S and DBT-SP interventions demonstrated a median frequency of self-harm ideation and behavior of 2 (i.e., one or more times per week) at baseline. At the 4-week follow-up after the intervention, the DBT-SP intervention showed superior effects compared with the DBT-S intervention (Supplementary Table 1). The time-by-group interactions for these outcomes showed a marginally significant trend (*p* = 0.07 and 0.08, respectively) (Table 3). For external emotion regulation and dissociation reversal, the time-by-group interaction *p* values were 0.05 and 0.07, respectively, also approaching statistical significance. Compared with the DBT-S intervention, the DBT-SP intervention was associated with greater reductions in anxiety and depression scores (*p* = 0.01 and 0.03, respectively). However, the time-by-group interaction for overall emotion regulation, as measured by the Emotion Regulation Scale (ERS), was not statistically significant (Table 3).

**Table 3:** Self-harm ideation, self-harm behavior, suicidal ideation, anxiety, depression, and emotion regulation among adolescents

Outcomes	DBT-S (median, IQR)				DBT-SP (median, IQR)				*p*- value		
	baseline	4 weeks	8 weeks	12 weeks	baseline	4 weeks	8 weeks	12 weeks	Interaction	group	time
NSSI thoughts	2 (1, 2)	2 (1, 2)	1 (1, 1)	1 (0, 1)	2 (1, 2)	1 (0, 1)	0 (0, 1)	0 (0, 1)	0.07	0.11	< 0.01
NSSI behavior	2 (1, 2)	2 (1, 2)	1 (1, 1)	0 (0, 1)	2 (1, 2)	1 (0, 1)	0 (0, 1)	0 (0, 1)	0.08	0.16	< 0.02
Functions of Non-Suicidal Self-Injury											
Emotion Regulations	15(12, 18)	12 (3, 16)	10 (4, 15)	10 (6, 13)	10 (3, 15)	12 (8, 16)	10 (5, 16)	8 (3, 16)	0.05	0.20	0.08
Interpersonal Effect	5 (2, 10)	4 (0, 7)	2 (0, 6)	3 (0, 4)	1 (0, 9)	6 (1, 14)	5 (0, 10)	2 (0, 7)	0.14	0.41	0.01
Self Punishment	3 (1, 4)	2 (0, 3)	2 (0, 2)	2 (0, 3)	1 (0, 4)	2 (0, 3)	1 (0, 3)	1 (0, 3)	0.23	0.80	0.14
Anti Dissociation	4 (3, 6)	3 (0, 4)	2 (1, 3)	2 (1, 3)	1 (0, 5)	5 (1, 6)	1 (0, 4)	2 (0, 5)	0.07	0.31	0.02
Suicidal ideation	11 (6, 17)	15 (6, 18)	15 (7, 19)	11 (4, 15)	7 (2, 16)	11 (7, 15)	5 (0, 12)	3 (0, 13)	0.41	0.08	0.05
Depressive symptoms	15(12, 17)	13 (11, 18)	11 (8, 14)	14 (9, 17)	16 (6, 20)	7 (5, 15)	5 (3, 9)	5 (2, 12)	0.21	0.03	< 0.01
Anxiety symptoms	12 (8, 16)	10 (7, 16)	8 (5, 12)	11 (5, 13)	11(4, 17)	10 (2, 10)	4 (2, 6)	2 (1, 7)	0.23	0.01	< 0.01
Emotion regulation											
Cognitive reappraisal	24 (20, 28)	24 (21, 27)	22 (16, 28)	24 (22, 29)	23 (16, 26)	24 (18, 27)	24(15, 27)	24(16, 26)	0.80	0.32	0.17
Expressive suppression	16 (13, 18)	16 (14, 18)	15 (12, 16)	16 (15,19)	13 (10, 17)	12 (10, 18)	16(10, 18)	16(7, 16)	0.33	0.10	0.18

No statistically significant differences were observed in the time-by-group interaction or between-group comparisons for parental anxiety, depression, emotion regulation, or parenting stress in either the DBT-S or DBT-SP interventions. Similarly, no significant differences were found in the time-by-group interaction or between-group comparisons for the Family Environment Scale (Table 4).

**Table 4:** Anxiety, depression, emotion regulation, parenting stress, family environment, and parent-child interaction among parents

Outcomes	DBT-S (median, IQR)				DBT-SP (median, IQR)				*p*- value		
	baseline	4 weeks	8 weeks	12 weeks	baseline	4 weeks	8 weeks	12 weeks	Interaction	group	time
Depressive symptoms	2 (1, 4)	2 (0, 5)	1 (0, 5)	1 (0, 1)	1 (0, 4)	2 (1, 5)	1 (0, 3)	1 (0, 2)	0.71	0.88	0.07
Anxiety symptoms	2 (1, 3)	1(0,2)	1(0,3.5)	0(0,2)	4(1,7)	3(1,7)	3(1,5)	2(0,5)	0.39	0.25	0.21
Emotion regulation											
Cognitive reappraisal	25 (22, 26)	26 (23, 27)	24(19, 27)	23(17, 26)	25(22, 28)	23 (19, 26)	24 (21, 25)	24 (22, 27)	0.07	0.93	0.77
Expressive suppression	17(15, 20)	18 (14, 20)	18(14, 19)	16(13, 17)	16 (14, 17)	16 (15, 18)	16 (15, 18)	16 (15, 17)	0.25	0.44	0.50
Family Environment and Function											
Cohesion	7 (5, 9)	8 (5, 9)	8 (6, 9)	7 (4, 9)	7 (3, 8)	7 (5, 8)	8 (6, 8)	7 (5, 9)	0.47	0.71	0.52
Expressiveness	6 (4, 8)	7 (4, 8)	7 (6, 8)	7 (5, 8)	6 (4, 7)	7 (5, 8)	7 (6, 7)	7 (5, 7)	0.97	0.97	0.09
Conflict	2 (2, 6)	3 (1, 5)	2 (2, 3)	2 (2, 2)	4 (3, 5)	5 (1, 5)	3 (2, 4)	3 (2, 4)	0.95	0.17	**< 0.01**
Independence	6 (5, 7)	7 (6, 8)	7 (7, 8)	7 (6, 7)	6 (5, 8)	7 (6, 8)	7 (7, 8)	7 (7, 8)	0.80	0.33	**0.02**
Achievement	4 (2, 6)	4 (3, 6)	4 (2, 6)	3 (2, 5)	5 (3, 6)	5 (3, 5)	4 (3, 5)	4 (3, 5)	0.85	0.33	0.36
Cultural	5 (3, 6)	4 (3, 6)	4 (3, 6)	5 (4, 7)	4 (2, 6)	4 (1, 5)	4 (2, 6)	5 (2, 6)	0.42	0.50	0.08
Recreational	6 (4, 7)	6 (5, 7)	7 (4, 8)	7 (5, 7)	6 (3, 7)	6 (4, 7)	6 (5, 7)	5 (4, 7)	0.43	0.37	0.36
Moral	3 (2, 4)	3 (3, 4)	5 (4, 5)	4 (2, 4)	4 (3, 6)	4 (3, 6)	4 (3, 5)	4 (2, 5)	0.09	0.61	0.07
Organization	5 (3, 7)	6 (4, 7)	7 (3, 8)	6 (4, 7)	5 (4, 7)	5 (4, 6)	6 (5, 7)	5 (4, 7)	0.93	0.86	0.84
Control	2 (1, 3)	2 (1, 3)	3 (1, 4)	3 (1, 4)	2 (1, 3)	2 (1, 4)	3 (1, 4)	3 (1, 5)	0.42	0.95	**0.04**
Parenting stress											
Parental Distress	31(25, 38)	29(24, 35)	32(23, 34)	27(18, 36)	32 (25, 43)	35 (25, 40)	33 (24, 38)	31 (26, 37)	0.89	0.59	**< 0.01**
Parent-Child Dysfunctional Interaction	28(23, 29)	26(23, 33)	27(22, 31)	24(17, 28)	31 (23, 39)	29(26, 38)	29(23, 36)	28 (24, 36)	0.98	0.23	0.09
Difficult Child	31(26, 36)	29 (25, 37)	30(22, 36)	25(21, 35)	33 (29, 36)	31 (28, 36)	27 (23, 34)	27 (26, 36)	0.33	0.75	**< 0.01**
Parenting stress total score	90(76, 106)	90(74, 101)	90(69, 98)	74(58, 95)	95(77, 126)	92(81, 114)	91(71, 110)	89(79, 106)	0.99	0.47	**0.04**

**Fig.2:**
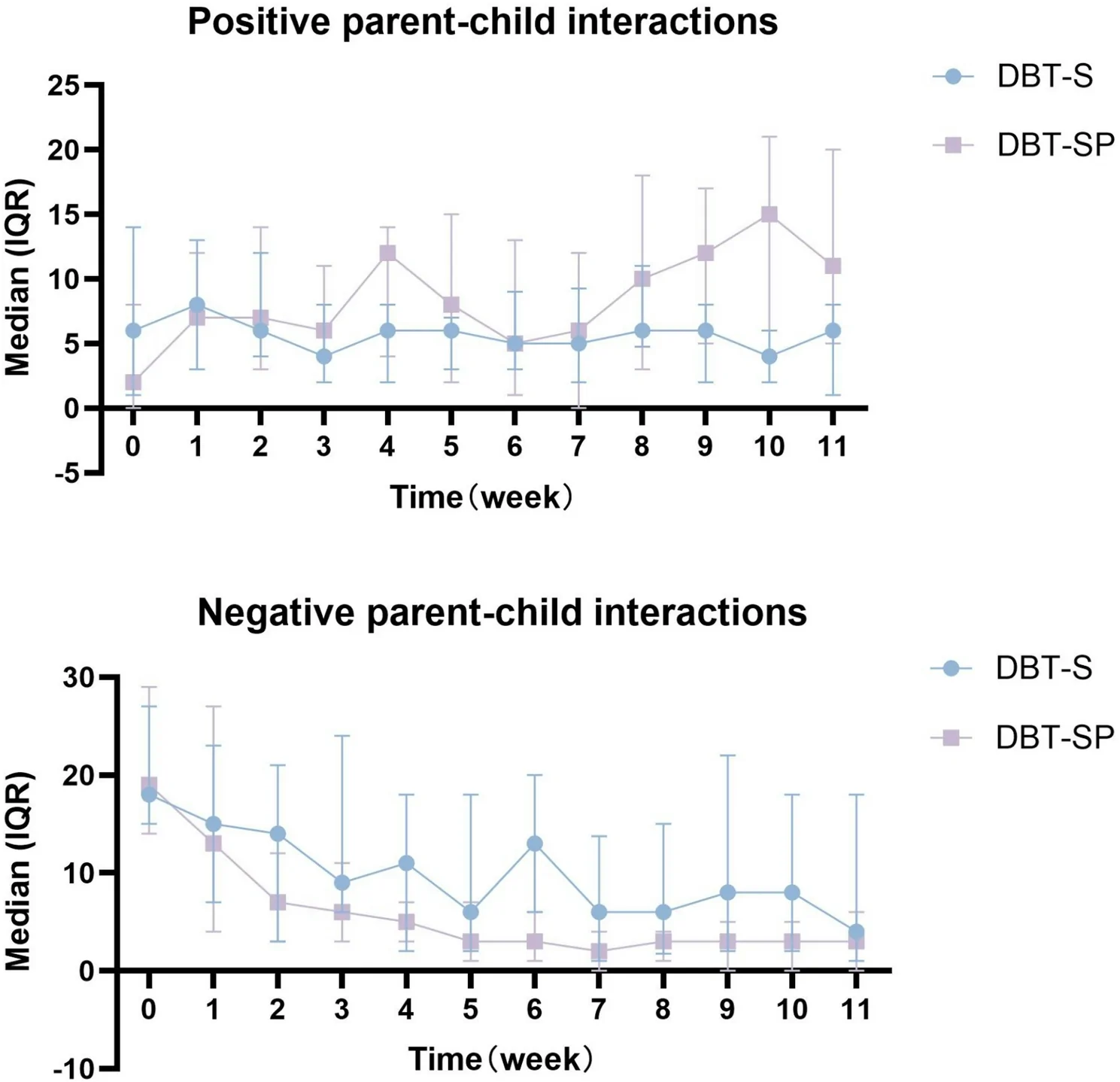
Weekly positive and negative parent-adolescent interaction frequencies across the 12-week intervention period. Median values with interquartile ranges (IQR) are shown for the DBT-SP group (purple squares) and the DBT-S group (blue circles) at the end of each weekly session, with week 0 representing baseline before the first session and weeks 1 through 11 representing post-session measurements following each successive intervention week. Panel A displays positive parent-child interactions; Panel B displays negative parent-child interactions. In the DBT-SP group, positive interactions increased from a median of 2 at baseline to 11 by the end of week 11, while remaining stable in the DBT-S group. Negative interactions decreased in both groups, with the DBT-SP group showing consistently lower levels from week 2 onward. Time × group interaction was significant for both positive and negative interactions (*p* < 0.01). Abbreviations: DBT-S, adolescent-only dialectical behavior therapy skills training; DBT-SP, parallel adolescent and parent dialectical behavior therapy skills training; IQR, interquartile range; W, week

Further analysis of parent-child interaction behaviors revealed that positive interactions in the DBT-SP intervention increased from a median of 2 times per week at baseline to 11 times per week by the end of week 11. In contrast, the frequency of positive interactions in the DBT-S intervention remained at a median of approximately 6 times per week from baseline to the end of week 11 (Table 5), with a significant time-by-group interaction (*p* < 0.01). Negative parent-child interactions decreased from a baseline median of 18 to 4 times per week by the end of week 11 in the DBT-S group, and from 19 to 3 times per week in the DBT-SP group, with a significant between-group difference (*p* < 0.01) (Fig. 2). For positive parent-child interactions, both the Interaction effect and the Time effect were statistically significant (*p* < 0.01). The main effect of group was not significant *(p* = 0.37). For negative parent-child interactions, the Interaction effect was not significant (*p* = 0.38) (Table 5).

**Table 5:** Association between treatment condition and parent-adolescent interaction outcomes

Outcomes	*p*- value		
	Interaction	Group	Time
Positive parent-child interactions	**< 0.01**	0.37	**< 0.01**
Negative parent-child interactions	0.38	**< 0.01**	**< 0.01**

Note. Bold values indicate *p* < 0.05. Descriptive data (median and IQR) for both groups are provided in Supplementary Tables 3 and illustrated in Fig. 2.

## Discussion

In this 12-week pilot randomized trial involving 38 adolescents with NSSI, both DBT-SP and DBT-S skills training were associated with substantial reductions in self-injurious behavior. The DBT-SP arm showed three preliminary signals: (1) earlier reductions in NSSI and larger reductions in co-occurring depressive and anxiety symptoms; (2) differential change in emotion regulation related functions of NSSI, in the absence of between-group differences in adolescents’ self-reported global emotion regulation strategies; and (3) a marked increase in positive parent-adolescent interactions in the DBT-SP arm, which may represent a preliminary relational marker of therapeutic change rather than a confirmed mediating mechanism.

The time-dependent reduction in NSSI across both treatment arms is consistent with prior evidence that structured DBT skills training alone may reduce self-harm [37, 38]. While adolescent DBT models theoretically emphasize caregiver involvement to enhance skills generalization and reduce invalidating family transactions, empirical support for its incremental benefit, particularly in brief skills-only formats, has been mixed [24, 37]. The present preliminary findings are compatible with the possibility that, when both groups receive identical skills curricula and show substantial improvement in primary outcomes, the added value of caregiver participation may be more readily observable in secondary outcomes such as co-occurring mood symptoms and family interaction patterns than in NSSI behavior itself. The parallel separate-group design evaluated here also accommodates pragmatic service delivery constraints and offers a feasible approach to incorporating caregivers without requiring conjoint sessions that some adolescents resist.

The convergence of (1) larger reductions in adolescent depression and anxiety, (2) differential change in emotion regulation related and anti-dissociation functions of NSSI, and (3) substantial improvements in parent-adolescent interaction patterns is consistent with a skills generalization account in which improvements may operate, at least in part, through the home environment. Concurrent caregiver skills training may increase the availability of real-time validation and coaching during episodes of emotional escalation, reduce criticism or conflict escalation, and enhance supportive exchanges that cue and reinforce adolescents’ use of distress tolerance and emotion regulation skills outside of sessions. As adolescents experience more effective interpersonal support and fewer invalidating transactions, their reliance on NSSI for emotion regulation or for interrupting dissociative states may diminish, a pattern that is broadly consistent with the functional changes observed at week 12 [24, 39, 40].

The absence of nominal between-group differences in caregiver-reported symptoms (depression, anxiety, parenting stress) may suggest that the primary mechanism of DBT-SP is not immediate reduction in caregiver psychopathology, but rather modification of how caregivers respond to adolescent distress [41, 42]. We note, however, that this interpretation is not the only possibility: the modest sample size, potential ceiling or floor effects on the caregiver-reported instruments, and limited sensitivity of these measures to short-term change over a 12-week window may also contribute to the null between-group findings. With these considerations in mind, the present data are compatible with, but do not establish, a behavioral pathway in which family involvement operates through improved family climate and parenting responses rather than solely through alleviation of caregiver mental health symptoms [11, 43].

## Strengths and Limitations

A strength of this trial is its parallel separate-group design, which enables concurrent caregiver skill acquisition without mandating conjoint sessions. This feature may enhance generalizability by including families characterized by high conflict or strained communication, populations often excluded from traditional conjoint DBT programs.

Several limitations warrant consideration. First, the absence of an attention-matched control condition means that the additional caregiver contact inherent in the DBT-SP arm may introduce nonspecific confounds (e.g., heightened family engagement, dose differences in total therapeutic contact between arms); future trials should incorporate a caregiver attention-control condition to isolate the specific effects of skills content. Second, the modest sample size (*n* = 19 per arm in the per-protocol analysis) constrained statistical power and resulted in wide confidence intervals for several effect estimates (e.g., NSSI odds ratios ranging from 1.38 to 52.32), and the per-protocol analysis itself reflected an attrition rate of approximately 33% from the originally randomized sample, which limits inference about the broader intent-to-treat population; adequately powered, multisite replication with pre-specified analysis plans is therefore a priority. Third, formal mediational analyses to examine whether parental DBT skills acquisition mediates improvements in adolescent NSSI and related outcomes were not performed, given the pilot nature and small sample size of the current study. Future well-powered trials using mediation designs are warranted to elucidate the underlying mechanisms of action. Fourth, the open-label design and exclusive reliance on self-report or caregiver-report measures may have introduced expectancy and social desirability biases, and the parent-adolescent interaction outcome in particular was reported by adolescents alone without multi-informant corroboration; subsequent studies should incorporate blinded clinician-rated assessments, multi-informant data, and ecological momentary assessment to mitigate these biases. Fifth, follow-up was confined to the immediate post-treatment period (week 12), and because the OSI assesses NSSI over the prior month, the week-12 measurement window partly overlapped with the active treatment period (weeks 9 to 12) rather than capturing a clearly post-treatment state; the durability of between-group differences therefore cannot be established from these data, and extended follow-up at 6 and 12 months is needed. Sixth, the trial was retrospectively registered after enrollment had begun; although the primary and secondary outcomes and the analytic plan were pre-specified in the study protocol prior to data collection, retrospective registration limits the external transparency of outcome pre-specification and is acknowledged as a constraint on the inferential strength of the present findings. Seventh, the exclusion of adolescents with severe acute suicide risk, severe depressive or anxiety symptoms requiring immediate intervention, or major comorbid conditions limits the generalizability of these findings to clinically more severe NSSI populations. Eighth, Parental marital status was imbalanced between groups at baseline. Although we adjusted for this variable in sensitivity analyses (Supplementary Tables 4 and 5) and found results consistent with the main analysis, post‑hoc adjustment does not fully address the imbalance. Hence, this limitation should be considered when interpreting the findings. In addition, the sample size (*N* = 38, 19 per group) is typical of a pilot study and provides limited statistical power. Consequently, confidence intervals for several effect estimates were very wide, indicating low precision. The non‑significant interaction terms (e.g., *p* = 0.08) should not be interpreted as evidence of no effect or as a trend. All findings are considered preliminary and require replication in a larger, adequately powered trial. Finally, parent-adolescent interactions were measured using a study-developed checklist that demonstrated acceptable internal consistency in this sample but lacks independent validation, and given the central role of this outcome in our interpretation, the corresponding findings should be regarded as preliminary; the instrument is provided in the Supplement to facilitate replication and psychometric evaluation in other populations. Given the pilot exploratory nature of this trial, no formal multiplicity correction was applied, all reported p values are nominal and uncorrected, and the findings should be interpreted as hypothesis-generating rather than confirmatory.

## Conclusions

This pilot trial provides preliminary evidence regarding the parallel-concurrent delivery of DBT skills training to adolescents with NSSI and their caregivers (DBT-SP) compared with adolescent-only delivery (DBT-S). Both formats were associated with substantial within-group reductions in core self-harm outcomes, while between-group differences in the pre-specified primary and secondary endpoints at week 12 did not reach nominal significance. Preliminary signals of larger reductions in negative parent-adolescent interactions and adolescent depressive symptoms in the DBT-SP arm may suggest that caregiver involvement could operate, at least in part, through relational pathways rather than directly altering NSSI behavior, although this interpretation remains tentative in the absence of direct mechanistic testing. Adequately powered, multisite trials with pre-specified analysis plans and extended follow-up are needed to examine whether these preliminary patterns replicate and to clarify the mechanisms through which family-inclusive DBT may confer benefit.

## Supplementary Information

Below is the link to the electronic supplementary material.

Supplementary Material 1

## Data Availability

All data generated or analyzed during this study are included in this published article and its supplementary materials.
