# Supplementary material for: Effect of parallel adolescent and parent dialectical behavior therapy skills training vs. adolescent-only skills training on nonsuicidal self-injury: a pilot randomized trial
Source: BMC Psychiatry. 2026 Jun 1;26:655. doi: 10.1186/s12888-026-08199-3 (PMC13505089; doi:10.1186/s12888-026-08199-3)
Supplement: Supplementary file 1 [file 12888_2026_8199_MOESM1_ESM.doc]

Supplementary Table 1. Effect of caregiver involvement on adolescent clinical outcomes: between-group effect estimates (β/OR with 95% CI).

| Outcomes | 4 weeks | 8 weeks | 12 weeks |
| --- | --- | --- | --- |
| NSSI-thoughts | 8.79 (1.48, 52.32) | 4.67 (0.73, 29.81) | 1.30 (0.21, 8.16) |
| NSSI-behavior | 8.33 (1.38, 50.23) | 5.13 (0.81, 32.26) | 1.50 (0.23, 9.66) |
| Functions of Non-Suicidal Self-Injury |  |  |  |
| Emotion Regulation | −0.23 (−5.36, 4.90) | −3.73 (−9.00, 1.54) | −5.63 (−9.27, −2.00) |
| Interpersonal Effect | 1.91 (−1.26, 5.08) | 0.73 (−2.37, 3.82) | −1.95 (−5.27, 1.38) |
| Self-Punishment | 0.30 (−1.00, 1.60) | −0.26 (−1.54, 1.01) | −1.05 (−2.22, 0.12) |
| Anti-Dissociation | −0.87 (−2.77, 1.03) | −0.62 (−2.22, 0.98) | −1.89 (−3.48, −0.31) |
| Suicidal ideation | −5.53 (−10.56, −0.50) | −3.58 (−8.32, 1.16) | −3.32 (−8.09, 1.46) |
| Depressive symptoms | −4.14 (−7.52, −0.76) | −4.79 (−8.78, −0.80) | −1.53 (−5.95, 2.89) |
| Anxiety symptoms | −4.60 (−7.62, −1.57) | −5.00 (−8.55, −1.45) | −1.84 (−5.65, 1.96) |
| Emotion regulation |  |  |  |
| ERS-CR | −0.78 (−6.31, 4.76) | −2.26 (−7.37, 2.84) | −3.00 (−7.68, 1.68) |
| ERS-ES | −0.30 (−3.75, 3.16) | −2.32 (−5.67, 1.03) | −2.42 (−5.25, 0.41) |

Abbreviations: NSSI, nonsuicidal self-injury; BSS, Beck Scale for Suicide Ideation; PHQ-9, Patient Health Questionnaire-9; GAD-7, Generalized Anxiety Disorder-7; ERS-CR, Emotion Regulation Scale–Cognitive Reappraisal; ERS-ES, Emotion Regulation Scale–Expressive Suppression; OR, odds ratio; CI, confidence interval.

Note. For NSSI-thoughts and NSSI-behavior, values are odds ratios (95% CI); for all other outcomes, values are β coefficients (95% CI) from linear mixed models.

Supplementary Table 2. Between-group effect estimates (β with 95% CI) for parent-reported outcomes.

| Outcomes | 4 weeks | 8 weeks | 12 weeks |
| --- | --- | --- | --- |
| Depressive symptoms | 1.45 (−1.08, 3.98) | 0.81 (−1.68, 3.31) | 1.02 (−1.55, 3.58) |
| Anxiety symptoms | 0.36 (−1.61, 2.34) | 0.90 (−1.05, 2.85) | −0.87 (−2.87, 1.13) |
| Emotion regulation |  |  |  |
| Cognitive reappraisal | 0.98 (−3.03, 4.98) | 1.93 (−1.72, 5.58) | 0.42 (−2.91, 3.75) |
| Expressive suppression | −0.28 (−3.05, 2.49) | 0.89 (−1.82, 3.60) | −1.79 (−3.89, 0.31) |
| Family Environment and Function |  |  |  |
| Cohesion | −0.51 (−2.17, 1.15) | 0.55 (−1.04, 2.13) | −0.74 (−2.39, 0.92) |
| Expressiveness | 0.17 (−0.96, 1.30) | 0.19 (−0.93, 1.30) | −0.02 (−1.17, 1.12) |
| Conflict | 0.49 (−0.45, 1.43) | 0.63 (−0.35, 1.61) | 0.42 (−0.99, 1.83) |
| Independence | 0.24 (−0.39, 0.87) | 0.56 (−0.30, 1.42) | 0.21 (−0.71, 1.13) |
| Achievement | −0.33 (−1.53, 0.87) | 0.15 (−1.04, 1.34) | −0.28 (−1.50, 0.94) |
| Cultural | −0.12 (−1.61, 1.37) | −0.66 (−2.27, 0.94) | −0.84 (−2.31, 0.62) |
| Recreational | −0.08 (−1.40, 1.24) | −1.02 (−2.36, 0.33) | −0.63 (−1.88, 0.62) |
| Moral | −0.54 (−1.51, 0.43) | −0.12 (−1.30, 1.06) | 0.84 (−0.22, 1.90) |
| Organization | −0.18 (−1.82, 1.47) | −0.19 (−1.62, 1.23) | 0.16 (−1.35, 1.66) |
| Control | −0.42 (−1.74, 0.90) | 0.15 (−1.05, 1.35) | −0.05 (−0.89, 0.78) |
| Parenting stress |  |  |  |
| Parental Distress | 1.03 (−3.75, 5.81) | 1.66 (−3.06, 6.38) | 1.61 (−3.23, 6.46) |
| Parent-Child Dysfunctional Interaction | 3.34 (−2.39, 9.07) | 2.85 (−2.74, 8.45) | 3.32 (−2.51, 9.15) |
| Difficult Child | −1.15 (−6.66, 4.36) | −0.14 (−5.91, 5.63) | 2.89 (−2.26, 8.05) |
| Parenting stress total score | 4.18 (−12.35, 20.71) | 4.85 (−10.72, 20.42) | 6.68 (−9.49, 22.86) |

Abbreviations: PHQ-9, Patient Health Questionnaire-9; GAD-7, Generalized Anxiety Disorder-7; ERS-CR, Emotion Regulation Scale–Cognitive Reappraisal; ERS-ES, Emotion Regulation Scale–Expressive Suppression; FES, Family Environment Scale; PD, Parental Distress; P-CDI, Parent–Child Dysfunctional Interaction; DC, Difficult Child; PSI-SF, Parenting Stress Index–Short Form; CI, confidence interval.

Note. Values are β coefficients (95% CI) from linear mixed models.

Supplementary Table 3. Parent–adolescent interaction frequencies at baseline and weeks 4, 8, and 12 by treatment group.

| Outcomes | DBT-S | | | | DBT-SP | | | |
| --- | --- | --- | --- | --- | --- | --- | --- | --- |
|  | Baseline | 4 weeks | 8 weeks | 12 weeks | Baseline | 4 weeks | 8 weeks | 12 weeks |
| Positive  parent–child interactions | 6 (1, 14) | 4 (2, 8) | 5 (2, 9) | 6 (1, 8) | 2 (0, 8) | 6 (4, 10) | 7 (2, 12) | 12 (5, 20) |
| Negative  parent–child interactions | 18 (15, 27) | 9 (6, 24) | 6 (1, 13) | 4 (1, 18) | 19 (14, 29) | 6 (4, 11) | 3 (1, 4) | 3 (0, 5) |

Abbreviations: DBT-S, adolescent-only dialectical behavior therapy skills training; DBT-SP, parallel adolescent and parent dialectical behavior therapy skills training. Data are presented as median (interquartile range).

| Supplementary Table 4. Self-harm ideation, self-harm behavior, suicidal ideation, anxiety, depression, and emotion regulation among adolescents (adjusted for parental marital status). | | | |
| --- | --- | --- | --- |
| Outcomes | *p*- value | | |
|  | Interaction | group | time |
| NSSI thoughts | 0.03 | 0.23 | <0.01 |
| NSSI behavior | 0.05 | 0.23 | <0.01 |
| Functions of Non-Suicidal Self-Injury |  |  |  |
| Emotion Regulatin | 0.05 | 0.37 | 0.08 |
| Interpersonal Effect | 0.14 | 0.54 | <0.01 |
| Self Punishment | 0.23 | 0.57 | 0.14 |
| Anti Dissociation | 0.07 | 0.31 | 0.02 |
| Suicidal ideation | 0.40 | 0.24 | 0.05 |
| Depressive symptoms | 0.21 | 0.10 | <0.01 |
| Anxiety symptoms | 0.23 | 0.04 | <0.01 |
| Emotion regulation |  |  |  |
| Cognitive reappraisal | 0.80 | 0.48 | 0.17 |
| Expressive suppression | 0.33 | 0.19 | 0.19 |
|  |  |  |  |

| Supplementary Table 5. Anxiety, depression, emotion regulation, parenting stress, family environment, and parent-child interaction among parents (adjusted for marital status). | | | |
| --- | --- | --- | --- |
| Outcomes | *p*- value | | |
|  | Interaction | group | time |
| Depressive symptoms | 0.71 | 0.88 | 0.07 |
| Anxiety symptoms | 0.39 | 0.25 | 0.21 |
| Emotion regulation |  |  |  |
| Cognitive reappraisal | 0.07 | 0.99 | 0.77 |
| Expressive suppression | 0.25 | 0.53 | 0.50 |
| Family Environment and Function |  |  |  |
| Cohesion | 0.47 | 0.81 | 0.53 |
| Expressiveness | 0.97 | 0.97 | 0.09 |
| Conflict | 0.95 | 0.26 | <0.01 |
| Independence | 0.81 | 0.17 | 0.02 |
| Achievement | 0.88 | 0.56 | 0.32 |
| Cultural | 0.41 | 0.84 | 0.08 |
| Recreational | 0.43 | 0.38 | 0.36 |
| Moral | 0.08 | 0.88 | 0.07 |
| Organization | 0.93 | 0.99 | 0.84 |
| Control | 0.42 | 0.98 | 0.04 |
| Parenting stress |  |  |  |
| Parental Distress | 0.88 | 0.59 | <0.01 |
| Parent-Child Dysfunctional Interaction | 0.98 | 0.69 | 0.08 |
| Difficult Child | 0.33 | 0.63 | <0.01 |
| Parenting stress total score | 0.98 | 0.91 | <0.01 |
|  |  |  |  |

Supplementary Table 6. Association between treatment condition and parent–adolescent interaction outcomes.

| Outcomes | *p*- value | | |
| --- | --- | --- | --- |
|  | Interaction | Group | Time |
| Positive parent–child interactions | <0.01 | 0.61 | <0.01 |
| Negative parent–child interactions | 0.38 | <0.01 | <0.01 |

**eAppendix 1**

**Adolescent–Parent 12-Week Interaction Frequency Scale**

Child’s Name: ______________	Date: ______________

*Instructions: Please record the frequency of each interaction item as accurately as possible over the past week. Each item is rated from 0 (did not occur) to 10 (occurred 10 or more times).*

| **Item** | **Content** | **Weekly Frequency** |
| --- | --- | --- |
| ***Positive Interaction Subscale*** | | |
| P1 | My parent showed understanding or empathy toward my emotional expressions. | 0–10 |
| P2 | My parent actively encouraged me to participate in family decisions. | 0–10 |
| P3 | I shared pleasant experiences or positive emotions with my parent. | 0–10 |
| P4 | My parent proactively offered help to resolve a current problem I was facing. | 0–10 |
| P5 | My parent and I engaged in enjoyable activities together (e.g., sports, games, outings). | 0–10 |
| P6 | My parent listened patiently to my thoughts or opinions. | 0–10 |
| ***Negative Interaction Subscale*** | | |
| N1 | My parent communicated with me in a negative or dissatisfied tone. | 0–10 |
| N2 | My parent criticized my behavior or performance. | 0–10 |
| N3 | I had arguments or emotionally intense conflicts with my parent. | 0–10 |
| N4 | My parent blamed or reproached me for not meeting their expectations. | 0–10 |
| N5 | My parent ignored or dismissed my emotional expressions or needs. | 0–10 |
| N6 | I experienced noticeable emotional tension or unpleasantness when communicating with my parent. | 0–10 |

*Note. Scores for each subscale are summed (range: 0–60), with higher scores indicating greater frequency of positive or negative interactions, respectively.*
